# Heterologous Expression of Argininosuccinate Synthase From *Oenococcus oeni* Enhances the Acid Resistance of *Lactobacillus plantarum*

**DOI:** 10.3389/fmicb.2019.01393

**Published:** 2019-06-21

**Authors:** Hongyu Zhao, Longxiang Liu, Shuai Peng, Lin Yuan, Hua Li, Hua Wang

**Affiliations:** ^1^College of Enology, Northwest A&F University, Yangling, China; ^2^Shandong Engineering and Technology Research Center for Ecological Fragile Belt of Yellow River Delta, Binzhou, China; ^3^Heyang Experimental and Demonstrational Stations for Grape, Weinan, China; ^4^Shaanxi Engineering Research Center for Viti-Viniculture, Yangling, China

**Keywords:** *Oenococcus oeni*, heterologous expression, acid stress, argininosuccinate synthase, *Lactobacillus plantarum*

## Abstract

*Oenococcus oeni* can survive well in wine (an acid-stress environment) and dominate malolactic fermentation (MLF). To demonstrate a possible role of argininosuccinate synthase gene (*argG*) in the acid tolerance response of *O. oeni*, a related *argG* gene was inserted into a plasmid pMG36e and heterologously expressed in *Lactobacillus plantarum* SL09, a wine isolate belonging to a species of relevant importance in MLF. The expression levels of the *argG* gene in *L. plantarum* were analyzed by RT-qPCR, argininosuccinate synthase (ASS) activity and cell properties (amino acids, pH, H^+^-ATPase activity, and ATP levels) were determined at pH 3.7 in comparison with that at pH 6.3. Results showed that the recombinant strain *L. plantarum* SL09 (pMG36e*argG*) exhibited stronger growth performance compared with the control strain (without *argG* gene), and the expression levels of *hsp1*, *cfa*, *atp*, the citrate and malate metabolic genes were apparently increased under acid stress. In addition, the recombinant strain exhibited 11.0-, 2.0-, 1.9-fold higher ASS activity, H^+^-ATPase activity and intracellular ATP level, compared with the corresponding values for control strain during acid-stresses condition, which may take responsible for the acid tolerance enhancement of the recombinant strain. This is the first work report on heterologous expression of *argG* gene, and the results presented in this study will be beneficial for the research on acid stress response of *O. oeni.*

## Introduction

*Oenococcus oeni*, an important lactic acid bacteria (LAB), is critical for winemaking owing to the ability of deacidification and stabilization of wine through malolactic fermentation (Betteridge et al., 2015; Peng et al., 2018; Romero et al., 2018). The optimal pH for *O. oeni* growth is 4.8–5.5, however, wine is a harsh environment, with high acidity (pH 3.0–3.5), which is considered as a major stress for *O. oeni* growth (Fortier et al., 2003; Rosi et al., 2003; Guzzo, 2011). *O. oeni* has a certain acid resistance mechanism because it survives well in a wine environment and play a crucial role in winemaking (Dimopoulou et al., 2018; Lorentzen and Lucas, 2019).

Significant efforts have been made in order to reveal the mechanism by which *O. oeni* tolerates acid stress. A potential method used by bacteria is the use of a H^+^-ATPase to pump H^+^ out of the cell and thus increase the acid tolerance (Fortier et al., 2003). The citrate metabolism affects the acid tolerance of *O. oeni*, owing to its end products (Augagneur et al., 2007), with Margalef-Català et al. (2016) finding that the genes involved in glutamine and glutamate metabolism were upregualted of *O. oeni* under stress. Darsonval et al. (2018) verified that the CtsR is the master regulator of stress-response in *O. oeni*. In addition, the arginine catabolism through the arginine deiminase (ADI) pathway is known to enhance acid tolerance by converting arginine into an alkaline product and by increasing external pH (Tonon et al., 2001; Arena and Manca de Nadra, 2005; Araque et al., 2016). Moreover, the *argG* and *argH* gene involved in acid tolerance response of *Lactobacillus casei* (Quivey et al., 2000), which were responsible for encoding argininosuccinate synthase (ASS) and argininosuccinate lyase (ASL), respectively. Of these, ASS is considered as the rate-limiting enzyme for arginine biosynthesis (Lemke and Howell, 2002), we observed that the *argG* gene of *O. oeni* SD-2a was apparently over-expressed after acid shock in our previous research (Liu et al., 2017), and functioned as a core regulatory gene during acid stress response according to its place in a gene co-expression network. However, the potential role of the *argG* gene on *O. oeni* acid stress has not been elucidated.

*Oenococcus oeni* seems to be good model for the study of mechanisms involved in stress resistance, but there are difficulties to genetically modify the *O. oeni* cells even using the current molecular biology technologies (Beltramo et al., 2004; Assad-Garcia et al., 2008; Darsonval et al., 2016). Currently, more attention was turned to other LABs as a model to explore *O. oeni*, Schümann et al. (2012) expressed *O. oeni mle* in *L. plantarum* WCFS1, and the recombinant *L. plantarum* cells expressing MLE accelerate the malolactic fermentation. Weidmann et al. (2017) reported that the production of Lo18 from *O. oeni* expressed in *L. lactis* improved tolerance to heat and acid stress, suggesting Lo18 may play a role in cytoplasmic protein and membrane stabilization during stress. These studies suggested that achieving heterologous expression in other LABs can be used to explore the stress response mechanism of *O. oeni*. In addition, *Lactobacillus plantarum* is able to conduct MLF, and some *L. plantarum* strains are commercially used MLF starter (Du Toit et al., 2011; Capozzi et al., 2012; Valdés La Hens et al., 2015; Berbegal et al., 2016).

Therefore, in the present work, the *argG* was heterologously expressed in *L. plantarum* and the expression of *argG* gene was detected by RT-qPCR and ASS activity. Moreover, the growth of recombinant (pMG36e*argG*) and control strain (pMG36e) were measured under acid stress (pH 3.0–4.0), and cell properties (amino acids, pH, H^+^-ATPase activity, and ATP levels) were also determined at pH 3.7 and 6.3 to study the heterologous expression of the *argG* gene in the recombinant *L. plantarum* strain.

## Materials and Methods

### Strains and Growth Conditions

The industrial *O. oeni* strain, SD-2a was isolated from a local wine region in Shandong Province, China (Wang et al., 2015), and preserved in China General Microbiological Culture Collection Center (CGMCC 0715). *L. plantarum* SL09 was isolated from red wine and identified by 16S rRNA gene analysis (Supplementary Table S1; Lee et al., 2016; Wang et al., 2016).

*Oenococcus oeni* SD-2a was cultured at 28°C in FMATB (5 g/L glucose, 5 g/L D, L-malate, 5 g/L yeast extract, 10 g/L peptone, 0.2 g/L MgSO_4_⋅7H_2_O, 0.05 g/L MnSO_4_⋅4H_2_O, 0.5 g/L cysteine/HCl, and 250 mL fresh tomato juice, pH 4.8). *L. plantarum* SL09 was cultured at 37°C in MRS broth, and *Escherichia coli* DH5α grew at 37°C in Luria-Bertani (LB) medium. Agar plates were prepared with 15 g/L agar. The culture of *L. plantarum* SL09 strain with plasmid pMG36e or pMG36e*argG* required the addition of erythromycin (Solarbio, Beijing, China), with a final concentration of 100 μg/ml, meanwhile, the *E. coli* DH5α strain with plasmid pMG36e or pMG36e*argG* required the addition of erythromycin (Solarbio, Beijing, China) with a final concentration to 200 μg/ml.

### Data Acquisition and Gene Screening

The changes in the transcriptome of *O. oeni* SD-2a during acid shock were studied previously (Liu et al., 2017), the RNA-seq data were downloaded from Sequence Read Archive (SRA) database with an accession number of SRP105332. The gene co-expression network was constructed in this study using the differential expression of genes and the Pearson model to calculate the co-expression coefficient and *P*-value between genes. Genes with a high co-expression coefficient and different expression levels (more than 2 folds) were selected.

### DNA Extraction and Plasmid Construction

**FIGURE 1:**
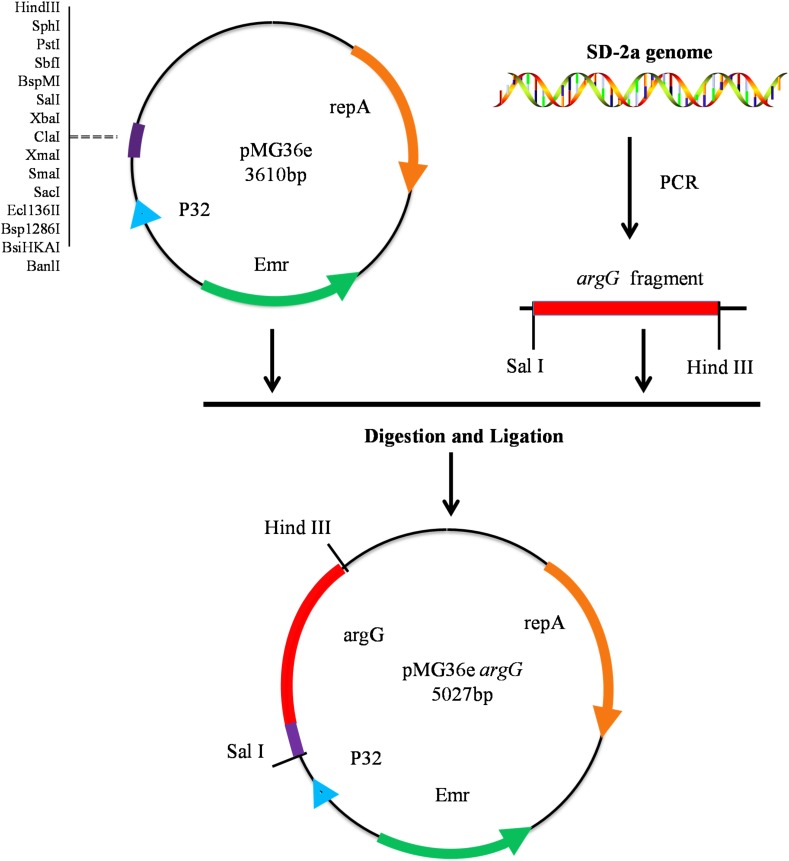
Construction of recombinant expression vector pMG36e*argG.*

*Oenococcus oeni* SD-2a was grown in FMATB medium to an OD_600__nm_ of 1.0 (5 × 10^8^ CFU/mL), then harvested by centrifugation at 12,000 × *g* for 2 min. DNA extraction was performed using the TIANamp Bacteria DNA Kit (Tiangen, Beijing, China) according to the manufacturer’s instructions. The *argG* gene from *O. oeni* SD-2a was amplified from genomic DNA using primers *argG*-F and *argG*-R (Table 1) to introduce the restriction site with PrimeSTAR^®^ HS DNA Polymerase (Takara). Subsequently, the PCR product and the vector pMG36e were digested by *Sal* I and *Hind* III. Both fragments were ligated and the resulting plasmid transformed into chemically competent *E. coli* DH5α cells according to the method recommended by the manufacturer (Takara) (Figure 1). Positive colony PCR amplified constructs were verified by sequencing, performed by a commercial provider, and the plasmid pMG36e*argG* was extracted using the Plasmid Mini Kit I (omega).

**TABLE 1:** Bacterial strains, plasmids, and primers used in this study.

**Strains, plasmids, or amplimer**	**Relevant property^a^**	**References/source**
*E. coli* DH5α	Cloning host	Takara
*O. oeni* SD-2a	Donor bacteria	Our lab
*L. plantarum*		
SL09	Plasmid-free bacteria	Our lab
SL09(pMG36e)	*L. plantarum* harboring pMG36e, Em^r^	This study
SL09(pMG36e*argG*)	*L. plantarum* harboring pMG36e*argG*, Em^r^	This study
Plasmids		
pMG36e	*E. coli*-*L. lactis* shuttle vector (3,6 kb), Em^r^	
pMG36e*argG*	pDL278-derivative vector containing the 1.4-kb region with the *argG* gene, Em^r^	This study
argG-F	CGCGGATCCGAAGGAGAA AAAATGGCAGATA	This study
argG-R	CCCAAGCTTGATCAGTCTA GCATGACCTG	This study

*^a^Em^r^, erythromycin resistance.*

### Cell Preparation and Electroporation

The overnight culture of *L. plantarum* SL09 was inoculated into 10 ml MRS broth supplemented with 4% glycine and incubated at 37°C to OD_600__nm_≈0.4. The cells were harvested by centrifugation, washed two times with 10 ml of sterile electroporation buffer (5 mM potassium phosphate, 0.5 mM MgCl_2_ and 0.5 M sucrose). Then, the cells were gently resuspended in 0.2 ml of electroporation buffer, and 100 μl of the solution was mixed together with 1 μg of plasmid DNA (pMG36e or pMG36e*argG*), transferred to a sterile 2-mm Gene Pulser cuvette (Bio-Rad) and left on ice for 5 min. Electroporation was performed with a Bio-Rad pulse gene controller (4 ms at 2.0 kV). The cells were immediately rescued into 1.8 ml of MRS supplemented with 0.3 M sucrose and incubated for 3 h at 37°C and plated onto MRS containing erythromycin (100 μg/ml). All strains and plasmids used in this study are listed in Table 1.

### Stress Challenges in *L. plantarum*

#### Stress Experiment

An overnight culture of SL09 (pMG36e*argG*) and SL09 (pMG36e) grown in MRS (pH 6.3) medium at 37°C was used to inoculate (1%, v/v) into fresh MRS media (pH 6.3), and cultured to an OD_600__nm_ of 1.0. The culture was then inoculated (1%, v/v, without washing treatment) into fresh normal MRS (pH 6.3) or acid-stressed MRS pH 3.0–pH 4.0 (gradient 0.1) then cultured at 37°C to investigated the growth performance of strains by measuring absorbance at 600 nm and counting plates colonies. The pH of the medium was adjusted by 1 M HCL using pH meter (INESA Scientific Instrument Co., Ltd., Shanghai, China). All growth experiments were carried out in triplicate, and all the culture added with 100 μg/mL erythromycin.

#### ASS Activity Assay and Determination of Amino Acids in Cells

The samples used in these assays were taken from cultures under pH 3.7 and pH 6.3, respectively. *L. plantarum* SL09 (pMG36e) and recombinant SL09 (pMG36e*argG*) were cultivated to logarithmic phase (8 h for pH 6.3, 36 h for pH 3.7). The cells were harvested by centrifugation at 12,000 × *g* for 10 min, washed twice with Tris–Hcl buffer, ground with liquid nitrogen, resuspended in Tris–Hcl buffer (50 mmol/L, pH 7.4), and then centrifuged at 12,000 × *g*, 4°C for 10 min to obtain the cell supernatant. Reaction mixtures included 100 μl of cell supernatant, 100 μl of 50 mM N-2-hydroxyethylpiperazine-N′-2-ethanesulfonic acid (pH 7.5), 16 mM ATP, 30 mM citrulline, 90 mM aspartic acid, and 5 mM MgCl_2_. Reactions were incubated at 27°C for 60 min and terminated with 70% (w/v) trichloroacetic acid. Final reaction supernatants were obtained by centrifugation at 5,000 × *g*, 4°C for 2 min. The supernatants added 300 μL O-phthalaldehyde and 600 μL borate buffer, then mixed at room temperature, filtered the mixture using 0.45 μm filtration, incubated in the dark strictly for 15 min, then applied to HPLC according to Hu et al. (2014) to obtain the concentration of other amino acids. Units of ASS activity (U) were expressed as micromoles of citrulline consumed per minute at 27°C (Cruz et al., 2007).

#### Measurement of pHi

Cells used for pHi measurement were cultivated to logarithmic phase (8 h for pH 6.3, 36 h for pH 3.7). The pHi was assayed by the fluorescence method using 2′, 7′-Bis-(2-Carboxyethyl)-5-(and-6)-Carboxy- fluorescein, acetoxymethyl ester (BCECF AM) as the fluorescent probe (Breeuwer et al., 1996; Zhang et al., 2007).

#### Determination of H^+^-ATPase and Intracellular ATP Concentration

Intracellular H^+^-ATPase activity was measured using the H^+^-ATPase assay kit (Beyotime Biotechnology, Shanghai, China). Enzyme activity units (U) were defined as the amount of enzyme required to oxidate 1 μmol NADH per minute at 37°C, pH 7.5. ATP concentrations were determined using a Firefly Luciferase ATP Assay Kit (Beyotime Biotechnology, Shanghai, China).

### RT-qPCR

Total RNA was extracted using the RNAprep pure Cell/Bacteria Kit (Tiangen, Beijing, China) following the manufacturer’s instructions. The quality of the RNA samples was verified on a 1% (v/v) agarose gel, and the concentration of RNA was determined by measuring the A_260__nm_ using a BioDrop μLITE Spectrophotometer (Tamar Laboratory Supplies LTD., Cambridge, United Kingdom). Next, cDNA was synthesized using the Thermo Fisher Scientific RevertAid First Strand cDNA Synthesis Kit (Thermo Fisher Scientific, MA, United States). RT-qPCR was conducted according to the instructions of ChamQ^TM^ SYBR qPCR Master Mix (Vazyme, Nanjing, China). The *L. plantarum* SL09 16S rRNA gene was used as the housekeeping gene, and the SL09 (pMG36e) served as the control strain and was cultivated at the same pH value (3.7 and 6.3) (Tang et al., 2017). The primers used for RT-qPCR are described in Table 2. The results were analyzed using the comparative critical threshold (2^–ΔΔCT^) method in which the amount of target RNA was adjusted to a reference signal (internal target RNA) as described previously (Livak and Schmittgen, 2001).

**TABLE 2:** Primers used for RT-qPCR.

**Gene**	**Primes Sequence (5’-3’)**	**Size**	**Description**	**References**
*argH*	CCGAAACGGGTGCTAAGTATG	135	Argininosuccinate lyase ASL	This work
	CAGCGGCAGGCAAAATACCAG			
*argF*	CCAGAGTTTTTGGGTAAGGAC	189	Ornithine carbamoyltransferase	This work
	CGTCGGGTGCCACTCATCGGT			
*cfa*	TTGGATGTTGGGAGTGGTTGG	123	Cyclopropane-fatty-acyl-phospholipid synthase	This work
	TTGCTTGATTTGCGCTTGTGT			
*hsp1*	TGGCACGCTCCTTCTGGGCAC	139	Heat shock protein	This work
	TCACGATAATCCAACTTCACA			
*uvrA*	ATTCCGATGGATGTGCCGTATG	148	UvrABC system protein A	This work
	TGATAACGCCCTCAAACACAGC			
*recA*	CCCCGTTTATGCGGAACACCTA	186	Protein RecA	This work
	GCGTCACCCATTTCACCTTCAA			
*recN*	CTGGTAAACGCACGAAAACGAG	94	DNA repair protein RecN	This work
	TTAGTGACTTTAGTTCCCGCCA			
*recF*	GGTTTATTTGGGTGTCTTGTCG	141	DNA replication and repair protein RecF	This work
	TTAATTGTTCCTGTTCTTGCGT			
*recO*	AACGCCCACTGAGTTCTGATAG	106	DNA repair protein RecO	This work
	TTCCGTGTTGGATTTGGCTAAG			
*mleA*	TAACCCCAGCCCAAAAAGC	279	Malolactic enzyme	This work
	TACCCGTGGCAACTAAGGC			
*mdh*	CAAGAACCTCGCAGGGATT	75	Malate dehydrogenase	This work
	ATTGTCGCCACCATTGCTG			
*mleP*	AATCTTGGCTAACGAAGCACAT	80	Malate permease	This work
	CCACGATGAACAACACGGTACT			
*atp*	GCGATGCTGTTCATTGCGAC	174	H^+^-ATPase	This work
	CGTTATCGTTCCCGTTTTGT			
*citP*	AAGCAATGGGCAGATGATGAGC	168	Citrate transport protein	This work
	AGCAACGAGTAGCAAGGAGACG			
*citE*	AAATGAAGAACGCTTACGGC	116	Citrate lyase	This work
	CGGCATCTTCCAAGTCAAAC			
*asnH*	TTACCGATTGGCACCCACAGT	125	Asparagine synthetase	This work
	ATTGCCAAGACTGAGACGGGG			
*aspB*	ATTCTGCCACCCCTGCTCGCC	131	Asparagine–oxo-acid transaminase	This work
	CGACGAATCAGTTTCAGGCAG			
*purA*	GCGTAGCCGACCTGCTTGATA	185	Adenylosuccinate synthetase	This work
	AATGACAACGGAAGTATCGGT			
*thrA*	ATTATCCATCCCGCTTCCACC	265	Aspartokinase/homoserine dehydrogenase 1	This work
	TCAACCACCCCACTACCCACA			
*glk*	ATCAAGCATTGGCACAGGTTT	129	Glucokinase	This work
	ACCCCACTACCCACAGTTCCT			
*pfk*	TGTTGTAGCCGTGTTCCGTTA	207	6-Phosphofructokinase	This work
	ACAAAATAGAAGCAGCCGACT			
*pgk*	AGCGTTGCATCATCGTTTCT	208	Phosphoglycerate kinase	In this study
	GCGCCGGAACAAATAACAAA			
*pycA*	AACTGTCAAAAATGCGGAAAA	171	Pyruvate carboxylase	This work
	CCTGGAATCGGCTGAAAGAAC			
*ldh*	ACATCGTTGTCATTACGGCTG	209	Lactate dehydrogenase	This work
	AATGACCCGATGCCGTGGAAA			
*gapdh*	TTCAGCAACCGACGATTCAA	194	Glyceraldehyde-3-phosphate dehydrogenase	This work
	AGCAGAAATCAAGACACGCT			
*argG*	GCGGTCTCATTGGATGTTGGC	252	Argininosuccinate synthase	This work
	GCTATGGCGACGGCATTATTA			
*16S rRNA*	AAGGGTTTCGGCTCGTAAAA	248	16S rRNA	This work
	TGCACTCAAGTTTCCCAGTT			

### Statistical Analysis

The activity of ASS and H^+^-ATPase, the concentration of amino acids, and the pHi and ATP levels were all determined in triplicate for each pH growth condition tested. A one-way analysis of variance (ANOVA) with Duncan test was performed using SPSS 19.0 software (SPSS Inc., Chicago, IL, United States) to investigate the significance of differences.

## Results and Discussion

### Gene Screening

**FIGURE 2:**
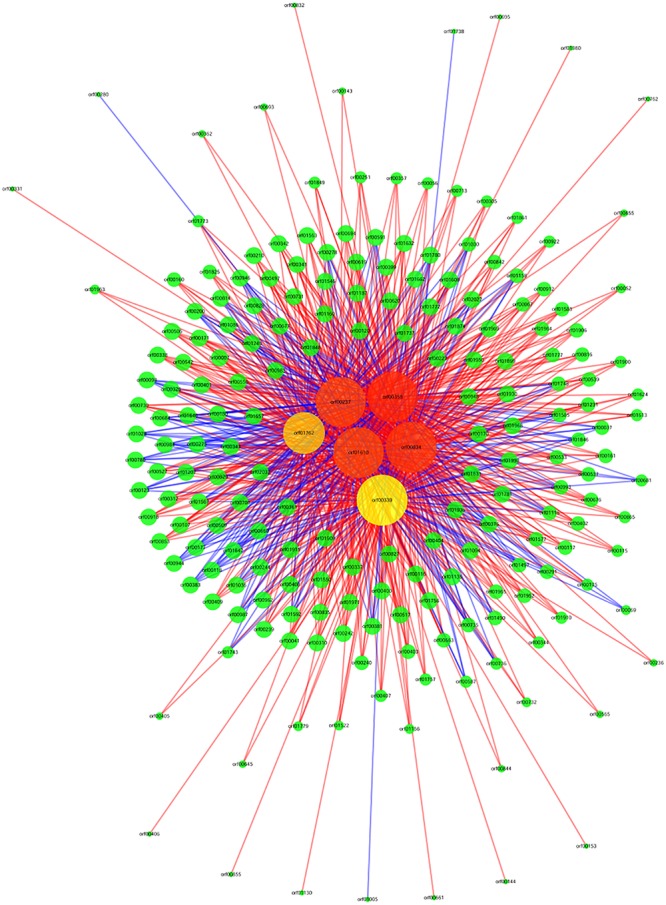
Gene co-expression network. Genes from *O. oeni* SD-2a after acid shock at 1 h and 0 h. Cycle nodes represent genes, the size of nodes represents the power of the interrelation among the nodes, and the edges between two nodes represent the interactions between genes. The more edges of a gene indicate that more genes connect to it, indicating it has a more central role within the network. A straight line represents a mutual relationship, red indicates that the correlation coefficient is positive, and blue indicates that the correlation coefficient is negative.

The *argG* gene (orf00834) is a core regulatory gene during acid stress response according to the co-expression network (Figure 2), and the *argG* gene was over-expressed (2.94 folds) after acid shock (pH 3.0) 1 h. This gene was selected for this assay.

### Growth Resistance of *L. plantarum* Under Different pH Conditions

**FIGURE 3:**
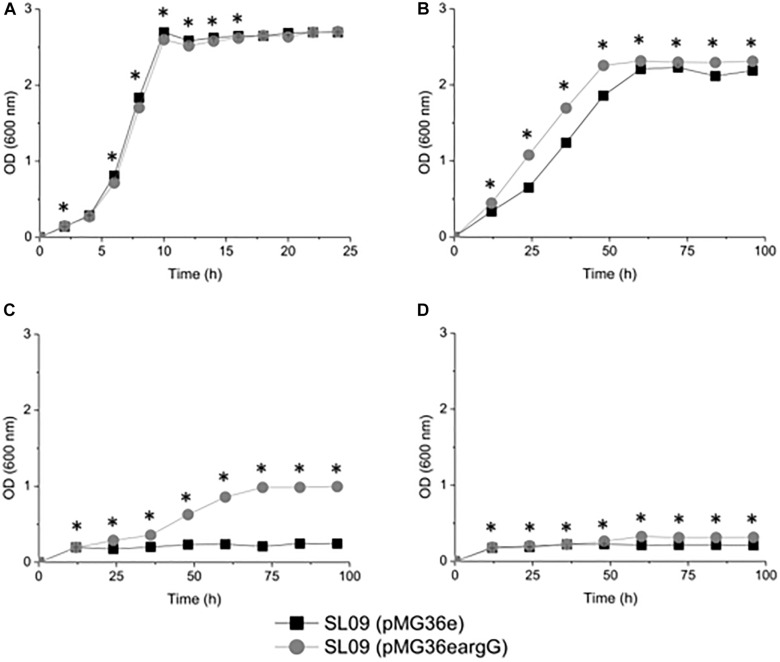
Growth curve of the recombination strain and the control strain under different pH conditions. **(A)** pH 6.3, **(B)** pH 3.7, **(C)** pH 3.3, and **(D)** pH 3.2. Values presented are the mean of three independent experiments. ^*^Difference significant at 95% confidence level.

To investigate the influence of ASS on the resistance of transformed cells toward acidity, the growth of each strain was evaluated at OD_600__nm_ and counted plates colonies under different pH conditions (Figure 3 and Supplementary Figure S2). Despite their similar growth performance at pH 6.3, the growth of recombinant strain *L. plantarum* SL09 (pMG36e*argG*) was more robust than that of the control *L. plantarum* SL09 (pMG36e) under acid stress conditions (pH 3.7, 3.3, and 3.2). As the pH decreased from 6.3 to 3.2, the maximum OD_600__nm_ of both strains gradually decreased. The maximum OD_600__nm_ of SL09 (pMG36e*argG*) was significantly higher than that of the control strain at pH 3.7. This difference was more obvious for the cells grown at pH 3.3, where the maximum OD_600__nm_ of the recombinant strain was 5-fold higher than that of the control strain. A similar result was also observed at pH 3.2, where only the SL09 strain (pMG36e*argG*) grew well. The results of plate counting shown similar performance as results of the OD_600__nm_ measurement.

The results indicated that the SL09 (pMG36e*argG*) strain displayed stronger resistance to acid stress than SL09 (pMG36e). Although the acidic environment still inhibited cell growth, the introduction of the *argG* gene dramatically enhanced the acid tolerance of *L. plantarum*, allowing these bacteria to survive at a lower pH, one which would normally reduce growth.

### ASS Activity Assay and Effect on Intracellular Amino Acids

**FIGURE 4:**
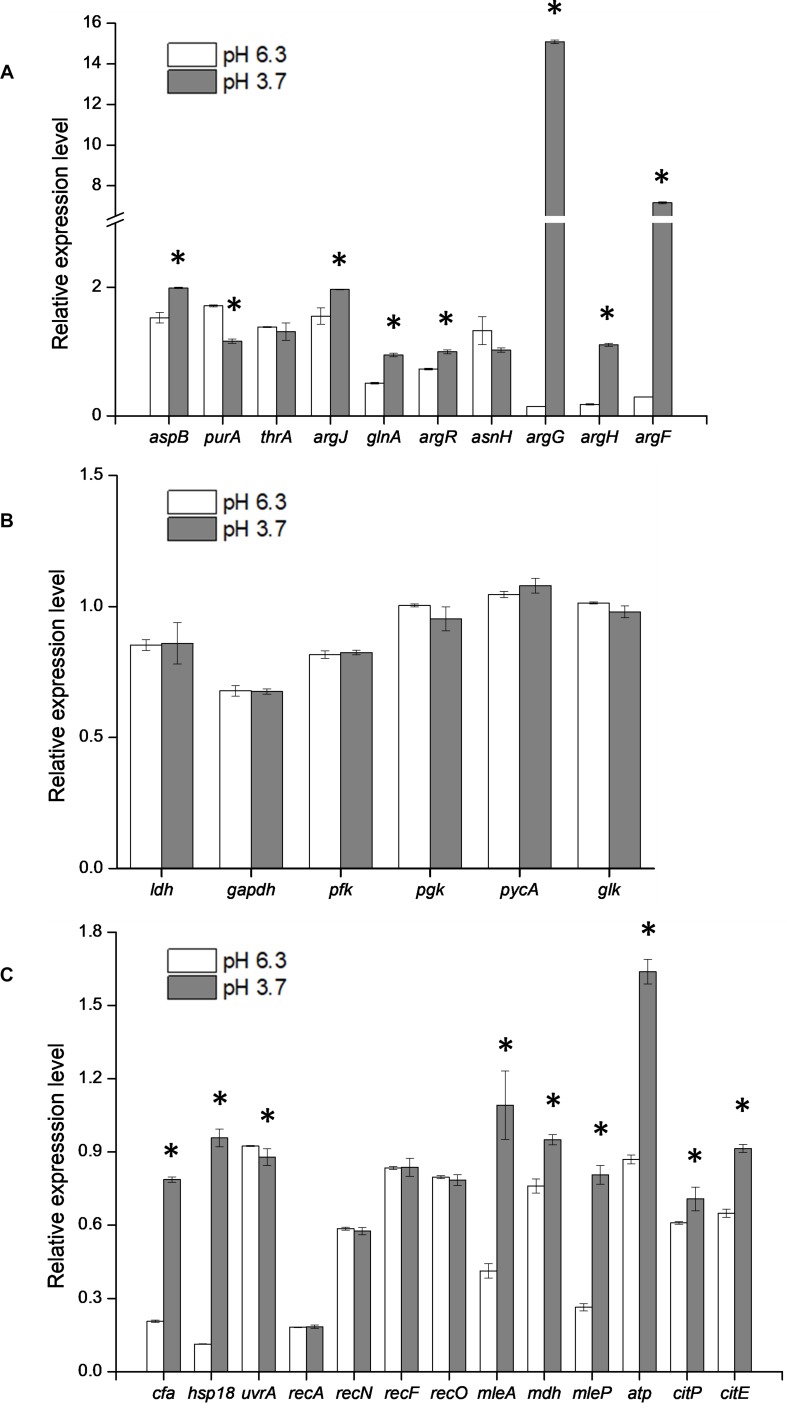
Effect of *argG* gene heterologous expression on the transcription of amino acid metabolic genes **(A)**, glycolytic genes **(B)**, and other stress response genes **(C)**. ^*^Difference significant at 95% confidence level.

**FIGURE 5:**
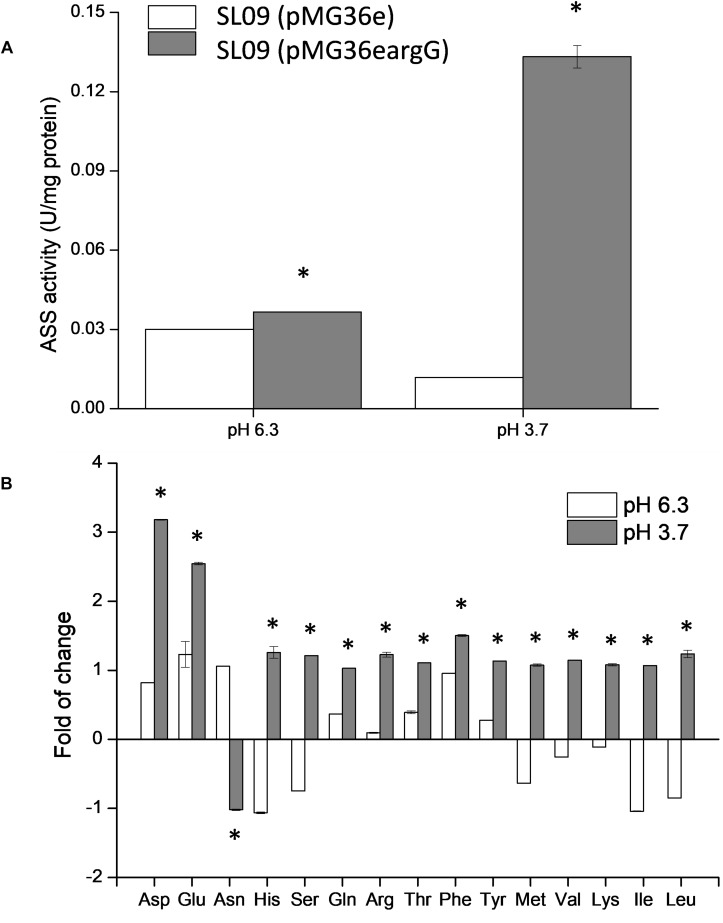
Effect of *argG* gene heterologous expression on ASS activity **(A)** and intracellular amino acids **(B)**. The fold change of intracellular amino acids in the recombinant strain were determined relative to the levels in SL09 (pMG36e) at pH 6.3 and pH 3.7. ^*^Difference significant at 95% confidence level.

To verify the heterologous expression of the *argG* gene, the transcriptional level of *argG* gene in recombinant and control *L. plantarum* was analyzed. (The RNA quality was shown in Supplementary Figure S1). As shown in Figure 4A, the expression level of *argG* was detected in the recombinant strain, (pMG36e*argG*) with strain SL09 (pMG36e) as control, and the relative expression level was significantly higher under acid stress conditions (pH 3.7). Figure 5A shows the ASS activity of both strains under the favorable and acid stress conditions (pH 6.3 and pH 3.7, respectively). Indeed, the recombinant strain exhibited higher ASS activity than did the control strain, especially under acid stress (pH 3.7, 11-fold difference). From pH 6.3 to pH 3.7, the ASS activity of the control strain was decreased by 61%, but the ASS activity of SL09 (pMG36e*argG*) increased by 260%. The improvement of ASS activity at pH 3.7 demonstrated that acid stress induced the high-efficiency expression of the *argG* gene in the recombinant strain. In arginine biosynthesis, ASS acts as the rate-limiting enzyme encoded by *argG* gene (Lemke and Howell, 2002). Indeed, as shown in Figure 5B, the amount of arginine synthesized was elevated, which may be attributed to the increased ASS activity level. Based on these findings, the acid tolerance enhancement of recombinant strain benefited from the heterologous expression of the *argG* gene that regulates ASS in the arginine deiminase pathway (ADI pathway).

Since the metabolism of amino acids is complex and consists of multiple interactions (Fernández and Zúñiga, 2006), the impact of heterologous expression of the *argG* gene on amino acid metabolic genes can be seen at the transcriptional level (Figure 4A). The expressions of *aspB*, *thrA*, *glnA*, *argR*, *argG*, *argH*, and *argF* were significantly higher when SL09 (pMG36e*argG*) was exposed to acid stress than under the control condition, while the expression of *purA* and *asnH* was decreased. We observed that the genes involved in the ADI pathway were upregulated while the genes converting aspartate into adenylosuccinate and asparagine were downregulated, which is beneficial to the accumulation of aspartate, an arginine precursor. Investigating further, the levels of intracellular amino acids in the recombinant strain were compared to those in the control strain at pH 6.3 and pH 3.7. As is shown in Figure 5B, the heterologous expression of *argG* gene increases the concentrations of aspartate, glutamate, glutamine, arginine, and threonine under acid stress, most of them are related to the ADI pathway, which was in accordance with the RT-qPCR results.

In this study, the heterologous expression of *argG* gene tilted amino acid metabolism toward ADI pathway, which can produce alkaline products to neutralize H^+^ (Tonon and Lonvaud-Funel, 2000), meanwhile, putrescine could be formed (Henríquez-Aedo et al., 2016). Putrescine is one of the biogenic amine present in wine, one which will affect the quality and safety of wine. The relationship of heterologous expression of *argG* gene and content of putrescine needs to be explored it next step. In addition, Bourdineaud et al. (2002) found that arginine stimulated pre-adaption of *O. oeni* to wine stress at the start of wine-making, which may be related to the expression level of stress response genes, including *ftsH*, *omrA*, and *arcR*, which were higher when the medium contained arginine. Subsequent study showed that arginine combined with fructose triggered the expression of *ftsH*, *omrA*, and *arcR* genes (Bourdineaud, 2006).

### ASS Effect on Glycolysis Pathway and Other Response Genes

Glycolysis is the major pathway that produces energy for LAB growth, except for the amino acid metabolism pathway. There are many genes involved glycolysis, such as the *gapdh*, *ldh, pfk*, *pgk*, *pycA*, and *glk* genes (Jan et al., 2013). Additionally, other stress response genes together with the genes in glycolysis pathway were chosen to investigate the influence induced by the expression of *argG* gene (Jobin et al., 1997; Grandvalet et al., 2008; Figures 4B,C). The expression level of all genes in this study was calculated based the expression of the control strain SL09 (pMG36e). Compared with pH 6.3, the expression level of some genes (*gapdh*, *ldh*, *pfk*, *pgk*, *pycA*, and *glk*) in glycolysis pathway were not significantly changed at pH 3.7. These results suggested that the heterologous expression of *arg*G in *L. plantarum* did not have significant effects on the glycolysis pathway under acid stress. This also recommended that those genes, such as *ldh*, can be used as internal control genes for RT-qPCR experiments in *L. plantarum* (Fiocco et al., 2008; Duary et al., 2010). Furthermore, at pH 3.7, the expression level of *cfa*, *hsp1*, *mleA*, *mdh*, *mleP*, *atp*, *citP*, and *citE* were higher than pH 6.3, displaying an increase between 1.0- and 8.5-fold. The cell membrane is the first barrier against an external unfavorable environment for LAB. Maintenance of the quality of the cell membrane is improved by the increased expression of the *cfa* and *hsp1* genes. In *O. oeni*, the CFA encoded by the *cfa* gene could reduce the effects of stress on the membrane, since cyclopropane rings restrict the overall mobility and disorder of acyl chains more than the *cis* double bonds. Additionally, in *O. oeni*, the *hsp18* gene encodes a small heat shock protein (sHSP), which contributes to the maintenance of membrane integrity under stress conditions by preventing the thermal aggregation of cellular proteins (Maitre et al., 2014). There are three genes which encode for small heat shock protein in *L. plantarum*. The *hsp1* gene was involved in controlling and improving membrane fluidity, and the *hsp3* gene may be responsible for the induction of thermotolerance. However, the deletion of *hsp2* did not significantly impair resistance to heat and other stresses (Arena et al., 2019). In this study, we investigated the expression level of *hsp1* genes which may be related to acid-stress response of *L. plantarum.* The consumption of H^+^ is another response to acid stress, and the recombinant strain at pH 3.7 showed a higher expression level of *atp* gene than pH 6.3. H^+^-ATPase is encoded by the *atp* gene, an enzyme can synthesize ATP using the H^+^ from the extracellular space into the cell.

The *mleP*, *mleA*, and *mdh* gene were important genes, responsible for malate metabolism (Augagneur et al., 2007, 2016), and *citP* and *citE* gene played a major role in citrate metabolism (Augagneur et al., 2007), the expression of these genes was enhanced and was beneficial in improving strain acid tolerance. The metabolism of L-malate and citrate does not directly provide an energy source, but decarboxylation and the efflux of metabolites generate a proton motive force that can be used to drive ATP synthesis by H^+^-ATPase (Olguín et al., 2010).

**FIGURE 6:**
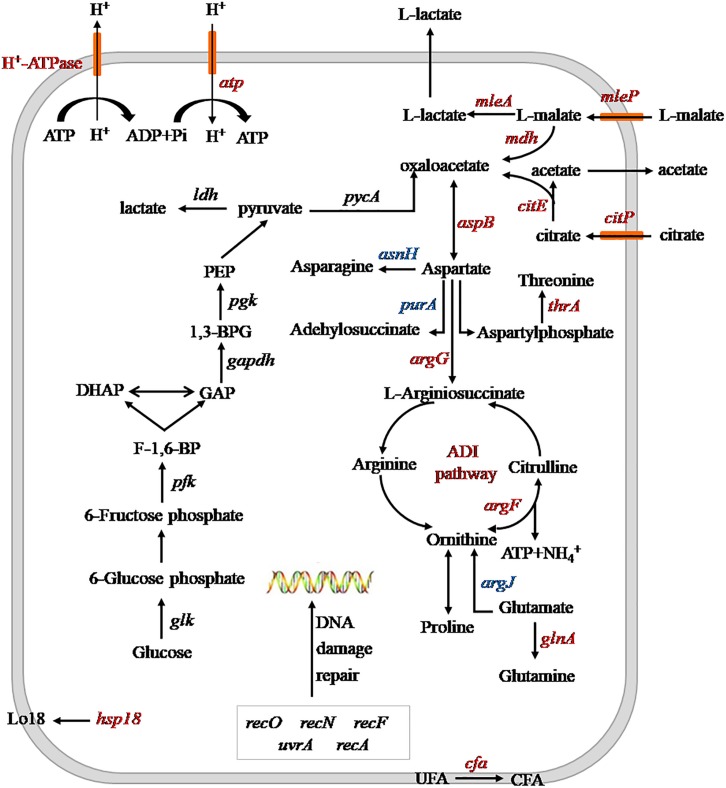
Schematic diagram of heterologous expression of the *argG* gene in SL09 at pH 3.7. Genes are in italics, the red color represents increased expression of genes or enzyme activity, the blue color represents decreased expression of genes. PEP, phosphoenolpyruvate; 1,3-BPG, 1,3- diphosphoglycerate; GAP, glyceraldehyde phosphate; DHAP, dihydroxyacetone phosphate; F-1,6-BP, fructose diphosphate; UFA unsaturated fatty acids; and CFA, cyclopropane fatty acids; ADI, arginine deiminase.

The effects of heterologous expression of the *argG* gene on SL09 at pH 3.7 are presented in Figure 6. The heterologous expression of *argG* gene did not affect the expression of genes related to DNA damage repair, because the *uvrA*, *recA*, *recN*, *recF*, and *recO*, were not significantly improved at pH 3.7,but may have stimulated the expression of *hsp1*, *cfa*, *atp*, and the malate and citrate metabolic genes under the acid condition. The functions of these genes include maintenance of the quality of the cell membrane, to exclude H^+^ and produce more ATP.

### ASS Effect on pHi, H^+^-ATPase Activity and Intracellular ATP Level

**FIGURE 7:**
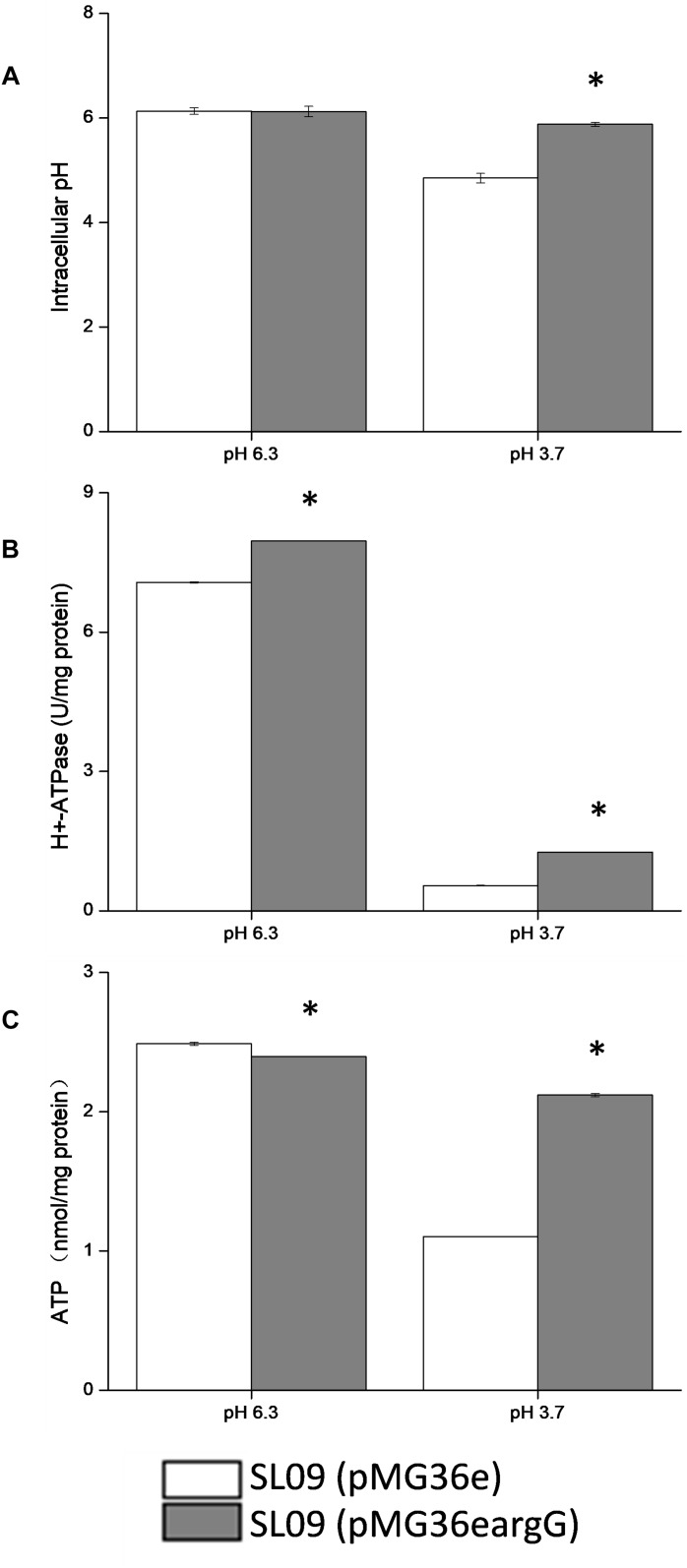
Effect of *argG* gene heterologous expression on intracellular pH **(A)**, H^+^-ATPase activity **(B)**, and intracellular ATP level **(C)**. ^*^Difference significant at 95% confidence level.

The pHi, H^+^-ATPase activity and intracellular ATP level of both strain were measured at pH 3.7 and pH 6.3, respectively (Figures 7A–C), to further investigate the effect of heterologous expression of the *argG* gene on acid stress resistance and the ASS effect on other stress response genes.

The intracellular pH of both strains did not show a significant difference at pH 6.3. Compared with pH 6.3, the pHi of the recombinant strain and the control strain were decreased at pH 3.7, the intracellular pH of the recombinant strain stabilized at 5.83, and the control strain decreased to 4.75. Obviously, the pHi of the recombinant strain declined less than the control strains. Although the H^+^-ATPase activity of both strains decreased at pH 3.7, but the H^+^-ATPase activity of the recombinant strain was two-fold higher than the control strain (pMG36e). Similarly, the ATP level of SL09 (pMG36e*argG*) and SL09 (pMG36e) were both decreased at pH 3.7, with the recombinant strain maintaining a higher ATP level (88.8%) than the wild-type strain (44.6%). These results were in accordance with the RT-qPCR results.

The intracellular pH affected a variety of biochemical reactions including reactions catalyzed by enzymes, since pHi is crucial for the maintenance of normal physiological activity in cells. The results indicated that the heterologous expression of the *argG* gene in *L. plantarum* increased the ability of cells to maintain neutral pH, which may be related to the higher mRNA level of *atp*, citrate and malate metabolic genes, and H^+^-ATPase activity under acid conditions. The pHi affected the activity of H^+^-ATPase, meanwhile the H^+^-ATPase activity also affected pHi because H^+^ is pumped out of cells through the H^+^-ATPase coupled with ATP hydrolysis (O’Sullivan and Condon, 1999). Moreover, citrate and malate metabolism could improve the pHi by using H^+^ during decarboxylation. The influence of the heterologous expression of the *argG* gene brought was complex and systematic and may increase the expression genes involved in consumption of H^+^ indirectly based on results, but further investigation into the relationship between them is needed.

In addition, the heterologous expression of the *argG* gene in *L. plantarum* contributed the ATP level in cells suggested by results. ATP levels in cells is a direct energy source, playing a crucial role in maintaining bacteria growth, proliferation, and cellular functions. The content of intracellular ATP was affected by many factors, in this study, the higher H^+^-ATPase activity of SL09 (pMG36e*argG*) under pH 3.7 promoted formation of ATP by consuming H^+^, the increased expression of genes involved malate and citrate of SL09 (pMG36e*argG*) under pH 3.7 was beneficial to ATP synthesis, and the heterologous expression of the *argG* gene in *L. plantarum* improved the concentration of amino acids participated in ADI pathway, which the arginine metabolism through the ADI pathway produces 1 mol of ATP per mol of arginine consumed. The level of intracellular ATP of SL09 (pMG36e*argG*) was higher than SL09 (pMG36e) according to the results, which will be helpful to the growth of the strain, the maintenance of cell functions, and the instigation of the stress mechanism. Therefore, the SL09 (pMG36e*argG*) demonstrated better performance than SL09 (pMG36e) under acid stress.

## Conclusion

The heterologous expression of *argG* gene from*O. oeni* SD-2a was achieved in *L. plantarum* SL09. Due to the expression of the *argG* gene, the acid stress resistance of recombination *L. plantarum* was improved, mainly affecting the ADI pathway, malate and citrate metabolism, with an increase in pHi, H^+^-ATPase activity and intracellular ATP levels. Additionally, the heterologous expression of *argG* gene also stimulated the expression of *hsp1*, *cfa*, and other genes related to malate and citrate metabolism, which requires further investigation. This work may be helpful to understand and eventually obtain *O. oeni* strains with high acid tolerance in winemaking industry.

## Data Availability

The datasets generated for this study can be found in Sequence Read Archive (SRA) database, SRP105332.

## Author Contributions

HL, LL, and HZ conceived the idea of the study. HZ, LL, SP, and LY designed and carried out the experiments. LL and HZ analyzed the data and wrote the manuscript. HW revised the manuscript.

## Conflict of Interest Statement

The authors declare that the research was conducted in the absence of any commercial or financial relationships that could be construed as a potential conflict of interest.
