# Supplementary material for: Heterologous Expression of Argininosuccinate Synthase From Oenococcus oeni Enhances the Acid Resistance of Lactobacillus plantarum
Source: Front Microbiol. 2019 Jun 21;10:1393. doi: 10.3389/fmicb.2019.01393 (PMC6598401; doi:10.3389/fmicb.2019.01393)
Supplement: Supplementary file 1 [file Table_1.DOC]

**Appendix A. Supplementary data**

**Table S1** 16sRNA gene analysis of the sequenced strain.

| Strain | Size(bp) | Relative strains | Identity | Accession number |
| --- | --- | --- | --- | --- |
| SL09 | 1461 | *Lactobacillus plantarum* | 99% | MF369877.1 |

**16sRNA sequence:**

CTCTGTCCTTAGGCGGCTGGTTCCTAAAGGTTACCCCACCGACTTTGGGTGTTACAAACTCTCATGGTGTGACGGGCGGTGTGTACAAGGCCCGGGAACGTATTCACCGCGGCATGCTGATCCGCGATTACTAGCGATTCCGACTTCATGTAGGCGAGTTGCAGCCTACAATCCGAACTGAGAATGGCTTTAAGAGATTAGCTTACTCTCGCGAGTTCGCAACTCGTTGTACCATCCATTGTAGCACGTGTGTAGCCCAGGTCATAAGGGGCATGATGATTTGACGTCATCCCCACCTTCCTCCGGTTTGTCACCGGCAGTCTCACCAGAGTGCCCAACTTAATGCTGGCAACTGATAATAAGGGTTGCGCTCGTTGCGGGACTTAACCCAACATCTCACGACACGAGCTGACGACAACCATGCACCACCTGTATCCATGTCCCCGAAGGGAACGTCTAATCTCTTAGATTTGCATAGTATGTCAAGACCTGGTAAGGTTCTTCGCGTAGCTTCGAATTAAACCACATGCTCCACCGCTTGTGCGGGCCCCCGTCAATTCCTTTGAGTTTCAGCCTTGCGGCCGTACTCCCCAGGCGGAATGCTTAATGCGTTAGCTGCAGCACTGAAGGGCGGAAACCCTCCAACACTTAGCATTCATCGTTTACGGTATGGACTACCAGGGTATCTAATCCTGTTTGCTACCCATACTTTCGAGCCTCAGCGTCAGTTACAGACCAGACAGCCGCCTTCGCCACTGGTGTTCTTCCATATATCTACGCATTTCACCGCTACACATGGAGTTCCACTGTCCTCTTCTGCACTCAAGTTTCCCAGTTTCCGATGCACTTCTTCGGTTGAGCCGAAGGCTTTCACATCAGACTTAAAAAACCGCCTGCGCTCGCTTTACGCCCAATAAATCCGGACAACGCTTGCCACCTACGTATTACCGCGGCTGCTGGCACGTAGTTAGCCGTGGCTTTCTGGTTAAATACCGTCAATACCTGAACAGTTACTCTCAGATATGTTCTTCTTTAACAACAGAGTTTTACGAGCCGAAACCCTTCTTCACTCACGCGGCGTTGCTCCATCAGACTTTCGTCCATTGTGGAAGATTCCCTACTGCTGCCTCCCGTAGGAGTTTGGGCCGTGTCTCAGTCCCAATGTGGCCGATTACCCTCTCAGGTCGGCTACGTATCATTGCCATGGTGAGCCGTTACCCCACCATCTAGCTAATACGCCGCGGGACCATCCAAAAGTGATAGCCGAAGCCATCTTTCAAGCTCGGACCATGCGGTCCAAGTTGTTATGCGGTATTAGCATCTGTTTCCAGGTGTTATCCCCCGCTTCTGGGCAGGTTTCCCACGTGTTACTCACCAGTTCGCCACTCACTCAAATGTAAATCATGATGCAAGCACCAATCAATACCAGAGTTCGTTCGACTTGCAGTATAGCACCCCCCA


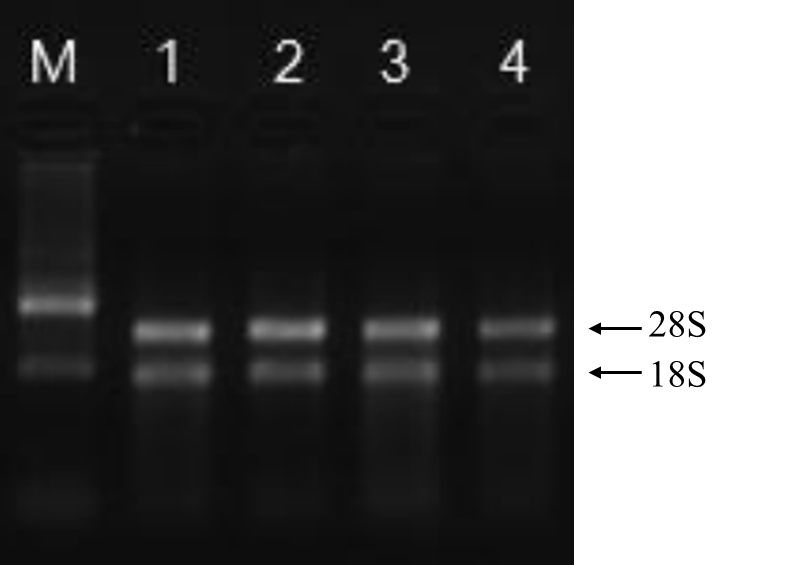


**Figure S1** The 1% agarose gel electrophoresis of RNA

1 represented the RNA extracted from SL09 (pMG36e) at pH 6.3, 2 represented the RNA extracted from SL09 (pMG36e*argG*) at pH 6.3, 3 represented the RNA extracted from SL09 (pMG36e) at pH 3.7, 4 represented the RNA extracted from SL09 (pMG36e*argG*) at pH 3.7.


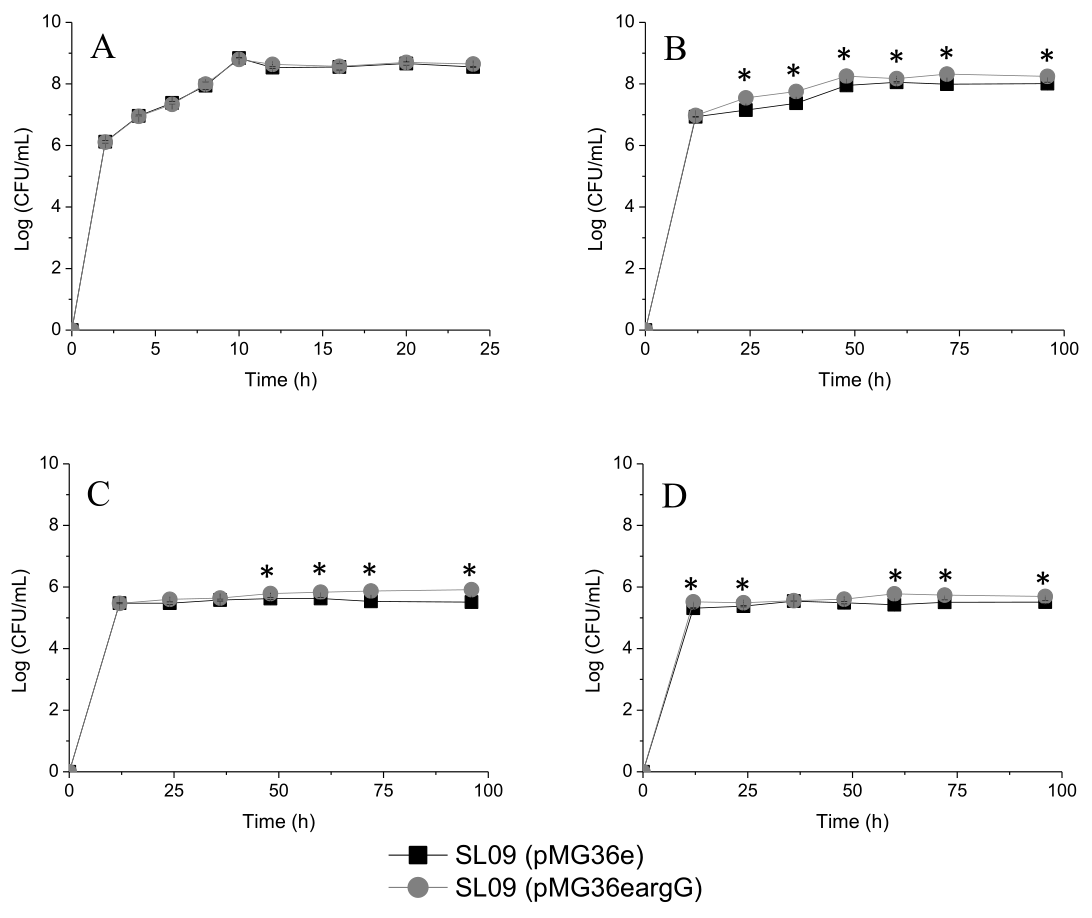


**Figure S2** Dynamics of strain biomass of recombination strain and the control strain under different pH conditions. A: pH 6.3, B: pH 3.7, C: pH 3.3, D: pH 3.2. Values presented are the mean of three independent experiments. *Difference significant at 95% confidence level.
